# Concurrent chemoradiotherapy with carboplatin followed by carboplatin and 5- fluorouracil in locally advanced nasopharyngeal carcinoma

**DOI:** 10.1186/1758-3284-3-30

**Published:** 2011-06-05

**Authors:** Tanadech Dechaphunkul, Kowit Pruegsanusak, Duangjai Sangthawan, Patrapim Sunpaweravong

**Affiliations:** 1 Department of Otorhinolaryngology Head and Neck Surgery, Faculty of Medicine, Prince of Songkla University, Songkhla, Thailand; 2 Division of Radiation Oncology, Department of Radiology, Faculty of Medicine, Prince of Songkla University, Songkhla, Thailand; 3 Division of Medical Oncology, Department of Internal Medicine, Faculty of Medicine, Prince of Songkla University, Songkhla, Thailand

## Abstract

**Background:**

This study aimed to evaluate acute major toxicities, the response rate, 3-year overall survival and progression-free survival rate of locally advanced nasopharyngeal carcinoma patients on concurrent carboplatin chemoradiotherapy followed by carboplatin and 5-fluorouracil.

**Methods:**

A prospective study of fifty patients diagnosed with locally advanced nasopharyngeal carcinoma received conventional radiation therapy with a total dose of 6600-7000 cGy in 6-7 weeks and concurrent chemotherapy of three cycles of carboplatin during radiotherapy, followed by adjuvant chemotherapy using carboplatin plus 5-fluorouracil for two cycles.

**Results:**

Weight loss and mucositis were the two most common acute major grades 3-4 toxicities (42%). Myelosuppression occurred subsequently, including leukopenia (30%), neutropenia (20%), anemia (12%), and thrombocytopenia (6%). Only 8% of patients developed grades 3-4 nausea and vomiting. No patients had renal and electrolyte abnormalities. Regarding the response evaluation, 100% of patients achieved an objective response rate of the primary tumor (92% complete response, and 8% partial response). Similarly, all patients also achieved an objective response rate of the neck node (64% complete response and 36% partial response). The 3-year overall survival rate and progression-free survival rate were 89.7% and 72.7%, respectively.

**Conclusions:**

Concurrent chemoradiotherapy with carboplatin followed by carboplatin and 5- fluorouracil could be considered as an alternative regimen for locally advanced nasopharyngeal carcinoma patients pertaining to a good overall response rate, 3-year overall survival and progression-free survival rate with good tolerability.

## Introduction

Nasopharyngeal carcinoma is a common head and neck cancer in Southeast Asia. The incidence is high in the third to fifth decade of life with a male predominance [1]. In Songklanagarind Hospital, there were approximately 80-100 new cases per year. Most of the patients were diagnosed in the advanced stage of the disease (60-70%).

According to the good response of radiotherapy (radiosensitive tumor) and anatomic restrictions, the standard treatment for nasopharyngeal carcinoma is definitive radiotherapy. However, 70% of patients presented with locally advanced stage cancer at the first time of diagnosis [2-5]. Moreover, it has been reported that nasopharyngeal carcinoma demonstrated the highest incidence of distant metastasis among all head and neck cancers [6-9]. The benefits of chemotherapy given concurrently with radiation are supported by the present data showing improvement in the effects of radiation through volume reduction, increased radiosensitizing effect, reduction of micro-metastasis, and overall survival improvement [10-12]. The first study was reported by the Head and Neck Intergroup 0099, using concurrent chemoradiotherapy with three cycles of cisplatin chemotherapy, followed by a 3-cycles combination of cisplatin and 5-fluorouracil chemotherapy. The results showed a significant benefit in both a progression-free survival and overall survival improvement [13]. Subsequently, there were several studies using the same combination of cisplatin and 5-fluorouracil chemotherapy as a first-line treatment in patients with nasopharyngeal carcinoma, and switching from cisplatin to carboplatin whenever the patients developed renal or serious toxicities from cisplatin [1,13-18]. Additionally, there were some studies using carboplatin as the first-line drug and the results showed no significant differences in the overall response rate and overall survival rate when compared to cisplatin [19-21].

Regarding toxicity profiles, carboplatin has less adverse events in terms of nausea, vomiting, renal toxicity and ototoxicity, but it does have a more myelosuppressive effect when compared with cisplatin. There is also no need for fluid hydration for carboplatin administration, thus the total infusion time is significantly shorter resulting in being a good choice of chemotherapy particularly in an outpatient setting.

Based upon the previous data, the objective of this study is to evaluate the benefit of carboplatin chemotherapy on response rate, 3-year overall survival rate, 3-year progression-free survival rate, including toxicity as a first-line treatment in combination with radiotherapy in patients with locally advanced nasopharyngeal carcinoma.

## Materials and methods

### 1) Patients

Fifty patients diagnosed with locally advanced nasopharyngeal carcinoma who received concurrent chemoradiotherapy during May 2005 to December 2007 at Songklanagarind Hospital were evaluated. Eligible patients were those with histologically confirmed nasopharyngeal carcinoma with at least T3 or N1 ≥ 3 cm or ≥ N2 as classified by the 6^th ^edition of the American Joint Committee on Cancer Staging System, and Eastern Cooperative Oncology Group (ECOG) performance status 0-2. The exclusion criteria included patients who were more than 65 years of age, patients previously receiving treatment for a locally advanced disease, recurrent or metastatic disease, inadequate hematological profiles (absolute neutrophil count < 1500/mm^3 ^or platelet count < 100,000/mm^3^), serum creatinine > 3 mg/dL, and poor compliance.

### 2) Study Procedure

All patients were evaluated by Head and Neck oncologist including complete history and physical examination, rigid nasopharyngoscopy, complete blood count, renal function, electrolyte and liver function tests. Radiological investigations included chest radiography and computed tomography or magnetic resonance imaging. An ultrasound of the hepatobiliary system was performed if there was a high level of serum alkaline phosphatase. This protocol was approved by the institutional ethics committees of Songklanagarind Hospital. All patients provided written informed consent.

**Figure 1:**
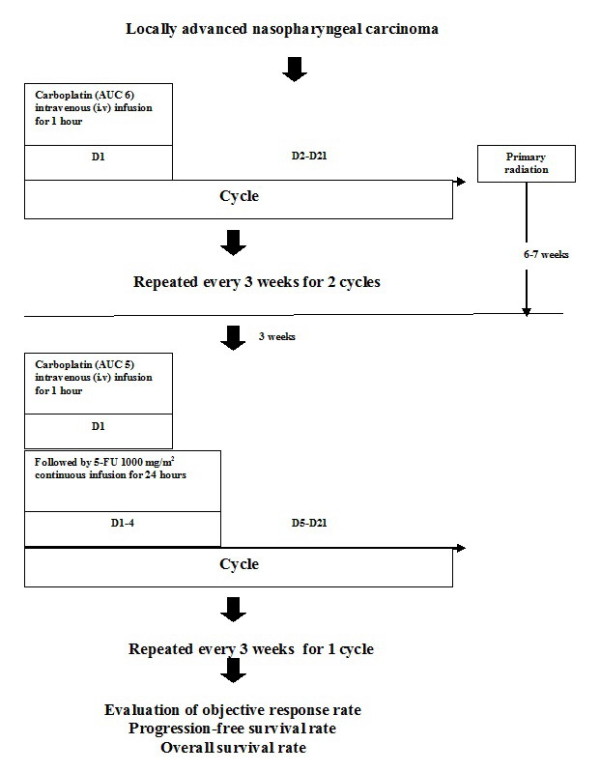
**Treatment protocol of concurrent chemoradiation**.

Conventional radiation therapy was used in all patients by 200 cGy per fraction with five daily fractions per week to a total dose of 6600-7000 cGy. A concurrent chemotherapy regimen was given by medical oncologist using carboplatin (AUC 6) intravenously every 3 weeks for 3 cycles during radiotherapy, followed by 2 cycles more of chemotherapy using carboplatin (AUC 5) plus 5- fluorouracil (1,000 mg per m^2 ^per day by 4 days infusion) every 3 weeks. The treatment protocol was shown in Figure 1. Dose modifications were performed according to patient's toxicity grading. Subsequent doses of chemotherapy were reduced by 20% if the patients developed grade 3 or 4 adverse events.

Patient's toxicities were graded by the Radiation Therapy Oncology Group toxicity criteria during treatment. The assessment of the tumor response was performed 3 weeks after the last cycle of adjuvant chemotherapy was completed including a complete physical examination, rigid nasopharyngoscopy and computed tomography or magnetic resonance imaging according to the Response Evaluation Criteria In Solid Tumor (RECIST) criteria. Patients were followed up every 2 months in the first year, every 3 months in the second year, every 4 months in the third year and then every 6-12 months until death. The treatment of a residual tumor was performed based upon the guidelines of our hospital.

### Statistical Analysis

In the statistical analysis, the epidemiology, clinical characteristics, and outcome data were assessed using the Fisher's exact test and the estimated survival probability was assessed with the Kaplan-Meier method by the R software package Epicalc.

## Results

Fifty patients diagnosed with locally advanced nasopharyngeal carcinoma were included in this study. All patients received concurrent chemoradiotherapy with a carboplatin agent.

The mean age of the patients was 44 years with a range of 22 to 62 years. The majority of patients were male (68%), AJCC stage III and IV (92%), and WHO histopathology type 1 (52%). Regarding cancer risk factors, only 16% of patients had a significant family history of cancer, however 50% of patients were active smokers and 44% were alcohol consumers. Most patients had an excellent ECOG performance status before treatment. Ninety-eight percent of patients could receive a completion of 5 planned cycles of chemotherapy. (Table 1)

**Table 1:** Patient Characteristics

Characteristics	Number of patients (%)
**Age**	44 (22-62)

**Sex**	

- Male	34 (68%)

- Female	16 (32%)

**Family history of cancer**	

- Yes	8 (16%)

- No	42 (84%)

**Active smoking**	

- Yes	25 (50%)

- No	25 (50%)

**Alcohol consumer**	

- Yes	22 (44%)

- No	28 (56%)

**Staging**	

- Stage IIb	4 (8)

- Stage III	18 (36)

- Stage IVa	19 (38)

- Stage IVb	9 (18)

**T stage**	

- T1	9 (18)

- T2a	3 (6)

- T2b	12 (24)

- T3	7 (14)

- T4	19 (38)

**N stage**	

- N0	2 (4)

- N1	9 (18)

- N2	30 (60)

- N3a	6 (12)

- N3b	3 (6)

**Histopathology (WHO)**	

- Type 1	26 (52)

- Type 2	1 (2)

- Type 3	23 (46)

**ECOG performance status**	

- 0	45 (90)

- 1	2 (4)

- 2	3 (6)

**Courses of chemotherapy**	

- 4 courses	1 (2)

- 5 courses	49 (98)

The most common symptom was a neck mass (82%). The other subsequent complaints included aural fullness (62%), nasal obstruction (42%), epistaxis (16%) and bloody sputum (14%).

**Figure 2:**
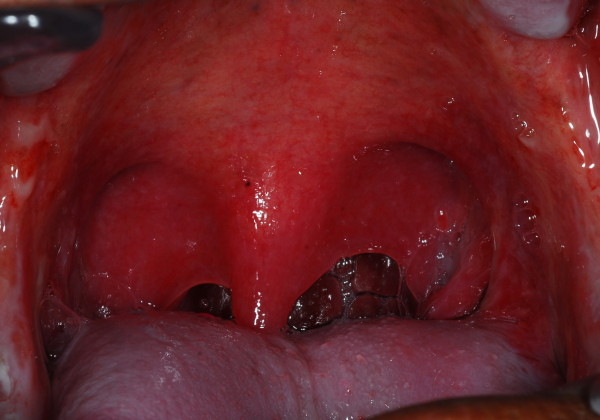
**Grade 1 mucositis, erythematous mucosa**.

**Figure 3:**
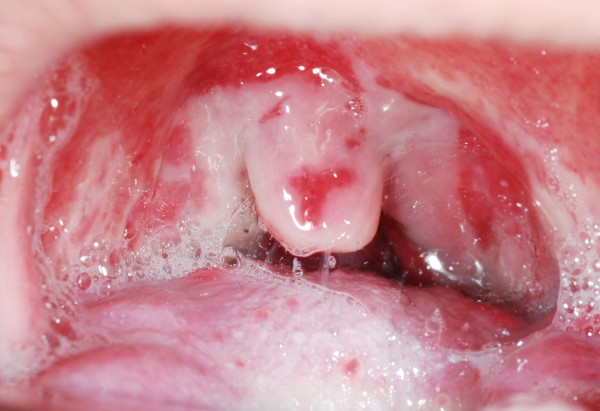
**Grade 3 mucositis, confluent pseudomembanous mucosa**.

Weight loss (42%) and mucositis (42%) were the most two common moderate to severe acute toxicities that was found in this protocol. Severity of mucositis was demonstrated in Figure 2 and 3. Myelosuppression occurred subsequently, including leukopenia (30%), neutropenia (20%), anemia (12%) and thrombocytopenia (6%). Only 8% of patients had moderate to severe nausea and vomiting and no patients had renal or electrolyte abnormalities from this treatment. (Table 2)

**Table 2:** Acute major toxicity profiles (N = 50)

Toxicity	Grading (No. (%))	
	
	Grade 1-2	Grade 3-4
	
	(Mild)	(Moderate to severe)
**Hematologic toxicity**		

Anemia	44 (88)	6 (12)

Thrombocytopenia	30 (60)	3 (6)

Leukopenia	31 (62)	15 (30)

Neutropenia	30 (60)	10 (20)

**Non-hematologic toxicity**		

Nausea	35 (70)	4 (8)

Vomiting	31 (62)	4 (8)

Mucositis	29 (58)	21 (42)

Fatigue	41 (82)	8 (16)

Weight loss	29 (58)	21 (42)

Skin	46 (92)	4 (8)

Hypokalaemia	29 (58)	0 (0)

Hyponatremia	15 (30)	0 (0)

Renal toxicity	2 (4)	0 (0)

Regarding the response evaluation, both the primary site and neck node had achieved an objective response rate of 100%. The primary site showed a 92% complete response and partial response was 8%. Sixty-four percent of neck node sites showed a complete response and 36% had a partial response. No patients had a stable and progressive disease. (Table 3)

**Table 3:** Response evaluation after complete treatment

Response evaluation	Number of patients (%)
**Primary**	

- Complete response	47 (94)

- Partial response	3 (6)

**Neck node**	

- Complete response	32 (64)

- Partial response	18 (36)

**Figure 4:**
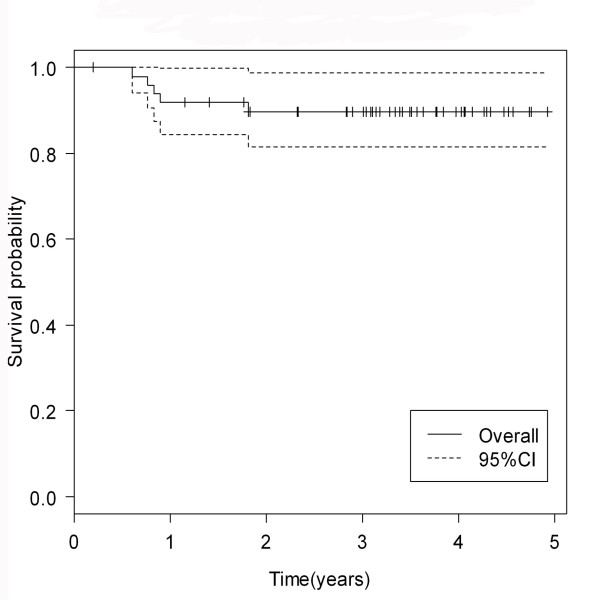
**3-year overall survival rate of locally advanced nasopharyngeal carcinoma patients**.

**Figure 5:**
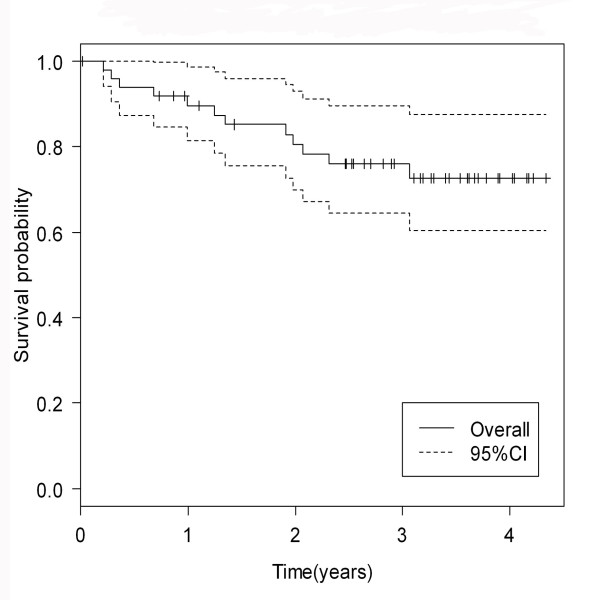
**3-year progression-free survival rate of locally advanced nasopharyngeal carcinoma patients**.

The median follow-up time was 37.3 months (range 3-59 months). (The 3-year overall survival rate and progression-free survival rate were 89.7% and 72.7%, respectively. (Figure 4, 5)

## Discussion

**Figure 6:**
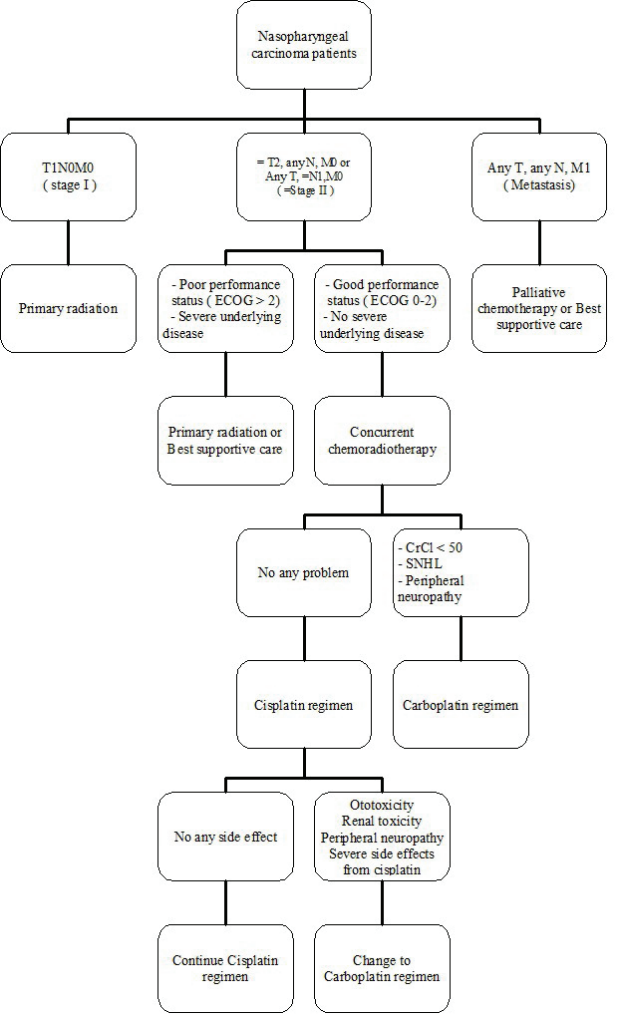
**Decision pathway for nasopharyngeal carcinoma patients**.

Nasopharyngeal carcinoma is a common head and neck cancer in Southeast Asia and more than 70% of patients presented with locally advanced stage cancer. According to the complexity of nasopharyngeal cancer treatment as well as the potential adverse events, a multidisciplinary approach is essentially encouraged. This requires the involvement of a head and neck oncologist, radiation oncologist and medical oncologist to achieve the best treatment outcome for individual patients. In our practice, the decision pathway is as shown in Figure 6.

Nowadays, concurrent chemoradiotherapy is the standard treatment for locally advanced nasopharyngeal carcinoma. Cisplatin chemotherapy was widely used as a first-line agent in combination with radiotherapy for nasopharyngeal carcinoma, whereas carboplatin would be considered as the option in case of intolerable side effects from cisplatin, especially ototoxicity and nephrotoxicity. Therefore, the objective of this study is to evaluate the benefit and safety profiles of carboplatin chemotherapy in combination with radiotherapy as a first-line treatment in these patients.

Our study strongly supported that carboplatin chemotherapy can be considered as a first-line alternative treatment for locally advanced nasopharyngeal carcinoma patients or switching treatment in patients who experienced the intolerable adverse events from cisplatin chemotherapy. There are many advantages in using carboplatin as a treatment in patients with locally advanced nasopharyngeal carcinoma. Firstly, our study showed comparable acute major toxicity between carboplatin and cisplatin chemotherapy, however there were less nausea, vomiting and renal impairment in favoring carboplatin chemotherapy. Mucositis was the most common acute major toxicity found in our study with 42% serious adverse events. Previous reports with cisplatin regimen showed mucositis was the most common acute major toxicity (61%), subsequently to leukopenia (32%), anemia (19%), thrombocytopenia (2%), vomiting (18%), renal impairment (2%) and hyponatremia (2%) [22]. However, thrombocytopenia was found to be more common in our study (6%). Thus, oncologists should be aware of this common adverse event and symptomatic treatment should be adequately offered to patients, which may eventually have an impact on the patients' compliance to therapy and their quality of life.

Secondly, our study with a carboplatin regimen showed good patient's compliance and tolerability. 98% of patients could receive a completion of 5 planned cycles of carboplatin chemotherapy. This is similar to another report which showed that the carboplatin regimen had a better tolerability than the cisplatin regimen [21]. Moreover, the carboplatin regimen took less time for chemotherapy infusion as there was no pre and post hydration. As a result, carboplatin could be considered the better option for outpatient cases.

Subsequently, carboplatin chemotherapy also demonstrated a comparable response rate to cisplatin. Data from previous literature on concurrent chemoradiotherapy with cisplatin regimen showed there was a wide range of responses between 49% to 98% [13,17,23-25]. In our study, the objective response rate of both the primary site and neck node showed 100%, however the complete response rate of the primary site was 92% and neck node site with a complete response showed only 64%. As such, carboplatin could be the acceptable alternative agent used for the treatment of nasopharyngeal carcinoma.

Finally, our data indicated a good overall survival and progression-free survival rate from concurrent chemotherapy with carboplatin chemotherapy with no substantially difference from cisplatin. The three-year overall survival rate and progression-free survival rate in nasopharyngeal carcinoma patients who received concurrent chemoradiotherapy with cisplatin were 71-89%, and 54-88%, respectively [10,17,21]. These were similar to our results showing 89.7% of a 3-year overall survival rate and 72.7% of a 3-year progression-free survival rate. Similarly, the recent data supported the benefit of carboplatin chemotherapy revealing no difference in the overall survival rate and progression-free survival rate between cisplatin and carbplatin chemotherapy given concurrently with radiation [21].

On the other hand, the nasopharyngeal carcinoma patients who had a residual neck node after treatment had to have performed salvage neck dissection according to the guidelines. However, there were some patients who needed to have their neck nodes observed and it was found that their neck nodes had subsided when follow up imaging was done 3-6 months later. Therefore, it may be that tumor evaluation should be extended to more than 3 weeks after the complete adjuvant chemotherapy for the actually tumor response or more accurate imaging such as PET-CT should be considered to decrease the likelihood of an unnecessary neck dissection.

In summary, our data strongly supported the benefit of concurrent carboplatin chemotherapy with radiotherapy for locally advanced nasopharyngeal carcinoma patients pertaining to a good overall response rate, 3-year over all survival and progression-free survival rate with good tolerability. It can be considered as an alternative first line optional therapeutic regimen for this patient group.

## Competing interests

The authors declare that they have no competing interests.

## Authors' contributions

TD carried out all data base collection, participated in study design, performed the treatment, did follow-up and statistical analysis. KP participated in its design, performed the treatment, and did follow-up. DS and PS performed the treatment, and did follow-up. All authors read and approved the final manuscript.

## References

[B1] Chan AT Teo PM Leung TW Johnson PJ The Role of Chemotherapy in the Management of Nasopharyngeal Carcinoma Cancer 1998 82 1003 1012 10.1002/(SICI)1097-0142(19980315)82:6<1003::AID-CNCR1>3.0.CO;2-F 9506343

[B2] Skinner DW Van Hasselt CA Nasopharyngeal carcinoma: methods of presentation Ear Nose Throat J 1990 69 237 240 2351084

[B3] Mackie AM Epstein JB Wu JS Stevenson-Moore P Nasopharyngeal carcinoma: The role of the dentistin assessment, early diagnosis and care before and after cancer therapy Oral Onco 2000 36 397 403 10.1016/S1368-8375(00)00034-8 10964045

[B4] Chen MS Lin FJ Tang SG Leung WM Leung W Clinical significance of cranial nerve deficit in the therapy of nasopharyngeal carcinoma Brit Radiol 1989 62 739 743 10.1259/0007-1285-62-740-739 2504432

[B5] Epstein JB Jones CK Presenting sign and symptoms of nasopharyngeal carcinoma Oral surg Oral med Oral pathol 1993 75 32 36 10.1016/0030-4220(93)90402-P 8419872

[B6] Chen WZ Zhou DL Luo KS Long-term observation after radiotherapy for nasopharyngeal carcinoma Int J Radiat Oncol Biol Phys 1989 16 311 314 10.1016/0360-3016(89)90320-9 2921131

[B7] Fandi A Altun M Azli N Armand JP Cvitkovic E Nasopharyngeal carcinoma:epidemiology, staging, and treatment Semin Oncol 1994 21 382 397 8209270

[B8] Vikram B Mishra UB Strong EW Manolatos S Patterns of failure in carcinoma of the nasopharynx: failure at distant sites Head Neck Surg 1986 8 276 279 3091536 10.1002/hed.2890080407

[B9] Zhang EP Lian PG Cai KL Chen YF Cai MD Zheng XF Guang XX Radiation therapy of nasopharyngeal carcinoma: prognostic factors based on a 10-year follow-up of 1302 patients Int J Radiat Oncol Biol Phys 1989 16 301 305 10.1016/0360-3016(89)90318-0 2921129

[B10] Afqir S Ismaili N Errihini H Concurrent chemoradiotherapy in the management of advanced nasopharyngeal carcinoma: Current status J Cancer Res Ther 2009 5 3 7 10.4103/0973-1482.48763 19293481

[B11] Baujat B Audry H Bourhis J Chan AT Onat H Chua DT Kwong DL Al-Sarraf M Leung SF Thephamongkol K Pignon JP MAC-NPC Collaborative Group Chemotherapy as an adjunct to radiotherapy in locally advanced nasopharyngeal carcinoma Cochrane Database Syst Rev 2006 18 CD004329 10.1002/14651858.CD004329.pub2 PMC9040103 17054200

[B12] Baujat B Audry H Bourhis J Chan AT Onat H Chua DT Kwong DL Al-Sarraf M Chi KH Hareyama M Leung SF Thephamongkol K Pignon JP MAC-NPC Collaborative Group Chemotherapy in locally advanced nasopharyngeal carcinoma: an individual patient data meta-analysis of eight randomized trials and 1753 patients Int J Radiat Oncol Biol Phys 2006 64 47 56 10.1016/j.ijrobp.2005.06.037 16377415

[B13] Al-Sarraf M LeBlanc M Giri PG Fu KK Cooper J Vuong T Forastiere AA Adam G Sakr WA Schuller DE Ensley JF Chemoradiotherapy versus radiotherapy in patients with advanced nasopharyngeal cancer: phase III randomized Intergroup study 0099 J Clin Oncol 1998 16 1310 1307 9552031 10.1200/JCO.1998.16.4.1310

[B14] Serin M Erkal HS Erkal Cakmak A Radiation Therapy and Concurrent Cisplatin in Management of Locoregionally Advanced Nasopharyngeal Carcinomas Acta Oncologica 1999 38 1031 1035 10665758 10.1080/028418699432310

[B15] Chua DT Sham JS Au GK Choy D Concomitant chemoirradiation for stage III-IV nasophayngeal carcinoma in Chinese patients: results of a matched cohort analysis Int J Radiat Oncol Biol Phys 2002 53 334 343 10.1016/S0360-3016(02)02710-4 12023137

[B16] Daniilidis J Fountzilas G Combined radiochemotherapy in locally advanced nasopharyngeal carcinoma HNO 2001 49 732 738 10.1007/s001060170045 11593775

[B17] Wee J Tan EH Tai BC Wong HB Leong SS Tan T Chua ET Yang E Lee KM Fong KW Tan HS Lee KS Loong S Sethi V Chua EJ Machin D Randomized trial of radiotherapy versus concurrent chemoradiotherapy followed by adjuvant chemotherapy in patients with American Joint Committee on Cancer/International Union against cancer stage III and IV nasopharyngeal cancer of the endemic variety J Clin Oncol 2005 23 6730 6738 10.1200/JCO.2005.16.790 16170180

[B18] Maoleekoonpairoj S Phromratanapongsa P Puttanuparp S Phase II study: Concurrent chemo-Radiotherapy in Advanced nasopharyngeal Carcinoma J Med Assoc Thai 1997 80 778 784 9470331

[B19] Paliament M Jha N Rapp E Smith C MacKinnon J Nabholtz JM Hanson J Reiman T Mackey J Concurrent weekly carboplatin and radiotherapy for nasopharyngeal carcinoma: report of a joint phase II study Radiother oncol 2001 58 131 136 10.1016/S0167-8140(00)00330-3 11166863

[B20] Okita J Hatta C Terada T Saeki N Ogasawara H Kakibuchi M Kamikonya N Sakagami M Concurrent chemo-radiotherapy for nasopharyngeal carcinoma Auris Nasus Larynx 2004 31 43 47 10.1016/j.anl.2003.09.004 15041053

[B21] Chitapanarux I Lorvidhaya V Kamnerdsupaphon P Sumitsawan Y Tharavichitkul E Sukthomya V Ford J Chemoradiation comparing cisplatin versus carboplatin in locally advanced nasopharyngeal cancer: Randomised, non-inferiority, open trial Eur J Cancer 2007 43 1399 1406 10.1016/j.ejca.2007.03.022 17467265

[B22] Lee AW Tung SY Chua DT Ngan RK Chapell R Tung R Siu L Nq WT Sze WK Au GK Law SC O'Sullivan B Yau TK Leung TW Au JS Sze WM Choi CW Fung KK Lau JT Lau WH Randomized Trial of Radiotherapy Plus Concurrent-Adjuvant Chemotherapy vs Radiotherapy Alone for Regionally Advanced Nasopharyngeal Carcinoma J Natl Cancer Inst 2010 102 1 11 10.1093/jnci/djq258 20634482

[B23] Lin JC Jan JS Hsu CY Liang WM Jiang RS Wang WY Phase III study of concurrent chemoradiotherapy versus radiotherapy alone for advanced nasopharyn-geal carcinoma: positive effect on overall and progression-free survival J Clin Oncol 2003 21 631 637 10.1200/JCO.2003.06.158 12586799

[B24] Chan AT Teo PM Ngan RK Leung TW Lau WH Zee B Leung SF Cheung FY Yeo W Yiu HH Yu KH Chiu KW Chan DT Mok T Yuen KT Mo F Lai M Kwan WH Choi P Johnson PJ Concurrent chemotherapy-radiotherapy compared with radiotherapy alone in locoregional advanced nasopharyngeal carcinoma: progression-free survival analysis of a phase III randomized trial J Clin Oncol 2002 20 2038 2044 10.1200/JCO.2002.08.149 11956263

[B25] Krstevska V Stojkovski I Klisarovska V Concurrent chemoradiotherapy in locally and/or regionally advanced nasopharyngeal carcinoma Prilozi 2008 29 295 307 19259054

